# Management of Diffuse Low-Grade Glioma: The Renaissance of Robust Evidence

**DOI:** 10.3389/fonc.2020.575658

**Published:** 2020-10-01

**Authors:** Karanbir Brar, Laureen D. Hachem, Jetan H. Badhiwala, Christine Mau, Brad E. Zacharia, Fabio Ynoe de Moraes, Farhad Pirouzmand, Alireza Mansouri

**Affiliations:** ^1^Faculty of Medicine, University of Toronto, Toronto, ON, Canada; ^2^Division of Neurosurgery, Department of Surgery, University of Toronto, Toronto, ON, Canada; ^3^Department of Neurosurgery, Penn State Health, Hershey, PA, United States; ^4^Penn State Cancer Institute, Hershey, PA, United States; ^5^Division of Radiation Oncology, Department of Oncology, Kingston General Hospital, Queen's University, Kingston, ON, Canada

**Keywords:** diffuse low-grade glioma, neurosurgery, randomized controlled trials, neuro-oncology, artificial intelligence, machine learning

## Abstract

The surgical management of diffuse low-grade gliomas (DLGGs) has undergone a paradigm shift toward striving for maximal safe resection when feasible. While extensive observational data supports this transition, unbiased evidence in the form of high quality randomized-controlled trials (RCTs) is lacking. Furthermore, despite a high volume of molecular, genetic, and imaging data, the field of neuro-oncology lacks personalized care algorithms for individuals with DLGGs based on a robust foundation of evidence. In this manuscript, we (1) discuss the logistical and philosophical challenges hindering the development of surgical RCTs for DLGGs, (2) highlight the potential impact of well-designed international prospective observational registries, (3) discuss ways in which cutting-edge computational techniques can be harnessed to generate maximal insight from high volumes of multi-faceted data, and (4) outline a comprehensive plan of action that will enable a multi-disciplinary approach to future DLGG management, integrating advances in clinical medicine, basic molecular research and large-scale data mining.

## Introduction

Diffuse low-grade gliomas (DLGGs, WHO Grade II gliomas), comprise 13–16% of all primary brain tumors (1). The median age at diagnosis is 37 years, and thus individuals are most commonly affected at the peak of their personal and professional lives. Progressive neurological decline and an early death are unfortunately inevitable (1). As such, there is a great need to optimize the management of these tumors in order to maximize survival while preserving quality of life.

The current DLGG management options after initial diagnosis include conservative therapy with radiographic monitoring, radiation therapy, or surgery, with or without chemotherapy adjuncts (2). Currently, one of the most contentious issues in the management of DLGG is the role of surgery and the importance of extent of resection on patient-related outcomes. There is a dearth of randomized-controlled trials (RCTs) or large prospective observational studies to support the benefit of increased extent of resection. While the traditional holy grail of evidence-based medicine is hypothesis-driven RCTs, there are a number of challenges in conducting an RCT for DLGGs. Questions of clinical equipoise and surgeon/patient-related biases, the continual influx of new adjuvant treatment discoveries, and the extended period of recruitment and monitoring needed to complete a trial are some of the many considerations that must be addressed prior to designing a robust RCT. In addition, the growth of big data and multi-centered patient registries have enabled the collection of valuable data in large quantities, which may offer new means to develop evidence-based management guidelines. With advancements in statistical methods and advanced data analytic concepts introduced by the field of machine-learning (ML), we now have powerful tools to develop better guidelines for the management of DLGGs and ultimately bring us closer to the concept of personalized medicine. The aim of this manuscript is to describe potential strategies to obtain new evidence for the management of DLGG, focusing on the integration of recent scientific advancements and alternative data sources with traditional methods such as the RCT.

## Current State of Evidence

The 2016 edition of the WHO classification of tumors has for the first time incorporated molecular parameters to traditional histological diagnoses of tumors (3). This is based in part on the impact of molecular features on prognosis and response to therapy (4–6). The genomic classification of tumors will soon supplant histology as a more accurate diagnostic method (7–9). Therefore, a large scale collaborative effort is necessary to better understand the prognostic/predictive role of known molecular markers (e.g., *MGMT* promoter methylation in DLGGs) (9), identify novel signatures, and generate hypotheses for future correlative clinical analyses.

Prior to the advent of molecular genetics in glioma profiling, biopsy was often deferred, with patients being followed with serial imaging even without biopsy. However, the profound impact of genomics on management has necessitated tissue acquisition; thus, biopsy has often been considered an essential step in the management of gliomas. In recent years, however, the surgical management of DLGGs has undergone a paradigm shift from the “wait and see” approach (with or without biopsy) to attempts at maximal safe resection (MSR) (10). The increase in overall survival (OS) and malignant progression-free survival (mPFS) observed in patients undergoing MSR has prompted calls for pursuing even more aggressive surgical resections (supratotal resections) wherein the limits of resection are defined by intraoperative functional brain boundaries rather than tumor margin or anatomical boundaries (11).

Although upfront MSR when feasible is endorsed by the latest European Association of Neuro-Oncology (EANO) guidelines (12), no Level I evidence exists to support this as standard of care (13, 14). Meta-analyses of observational studies demonstrate the benefit of increased extent of resection (EOR) on survival, but the yield of these studies is only as good as the primary data (15). In a parallel cohort analysis of outcomes for DLGG patients managed at two institutions in Norway, Jakola et al. have provided the strongest observational evidence to date favoring upfront surgery (16). A long-term follow-up of this study, adjusting for molecular markers, has reaffirmed this advantage (17). Correlation of EOR based on tumor location and molecular pathology was not provided. In another single-arm retrospective study of 228 adults with supra-tentorial DLGGs, it was shown that any incremental increase in the EOR is associated with increase in OS, regardless of molecular subclass (18). The sample in this study was enriched for tumors in the frontal (53.1%) and temporal (16.2%) lobes; only 39.5% of patients had tumor in an eloquent location (18). While these studies provide strong evidence for surgery, further exploration is needed.

A higher proportion of midline DLGGs is constituted by astrocytomas (19). In addition, a significantly higher proportion of IDH-*mutant* tumors, which are categorically more amenable to a complete resection, are located in the frontal lobe (7, 20). Hence, the interaction of subtotal resection with an unfavorable molecular/histological profile cannot be ruled out. Furthermore, although feasible, MSR of tumors in eloquent regions has most often been reported by specialized high-volume surgical centers (21). Other centers and clinical teams may not be as well-versed with the utility and interpretation of available diagnostic adjuncts and intraoperative techniques.

With respect to diffuse gliomas, surgery is not curative (10). While MSR may delay the need for toxic adjuvant therapy, objective assessment of the risks and benefits of this philosophy is needed, especially since practice-altering adjuvant therapies such as combination chemotherapy and radiotherapy have demonstrated a significant impact on OS in high quality RCTs (4–6, 22). Before developing similar high-quality evidence for surgery in DLGG, it is important to establish whether a surgical RCT can be justified on the grounds of equipoise and ethics.

## Toward a Possible RCT in DLGGs

### RCTs, Equipoise, and Ethics

**Figure 1:**
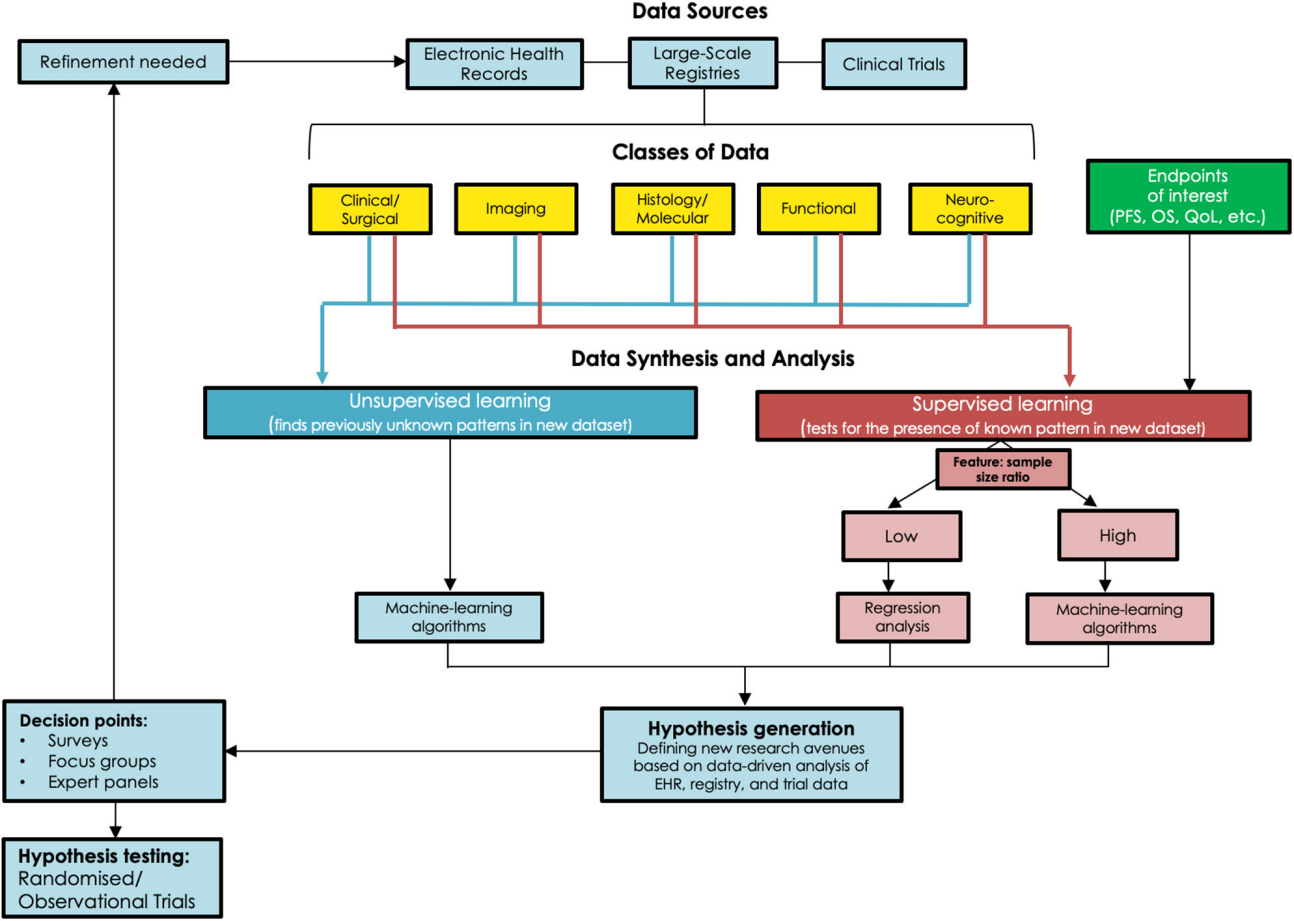
Schematic outlining the various strategies to acquire new data in the management of DLGGs and generate hypotheses for further research, and the need for observational or randomized trials to validate these hypotheses.

Equipoise, both within the medical community and among individual physicians, is an ethical necessity for RCTs (23). For surgical interventions, the timing of a trial adds a further layer of complexity to the preservation of equipoise. A technique in its infancy may be prematurely deemed ineffective while a well-established technique may reduce the chances of patient and surgeon participation, a conundrum that is very applicable to DLGGs (24). In a survey of 87 Canadian neurosurgical surgeons and trainees, 94% endorsed not knowing what the right treatment would be for DLGGs (2). As recently as 2011, half of the 24 neurosurgical centers surveyed in Germany implemented the “wait and see” approach routinely (25). A recent survey of Society for Neuro-Oncology members showed that nearly half of participants would consider an RCT to be beneficial for determining the differing roles of biopsy, surgery, and observation, particularly in certain patient populations (26). Although these data are derived from selective survey studies, the degree of uncertainty and heterogeneity in management is suggestive of possible clinical equipoise in certain patient populations. Experienced clinical teams are less likely to believe in equipoise and the ethical nature of a surgical RCT for managing DLGG patients. This poses a great challenge to advancing the state of evidence as calls for transitioning care for these complex patients to high-volume specialty centers increase (12). To better objectively assess whether true equipoise exists for any patient populations, surveys of international neuro-oncology societies, focus groups, and opinions of expert panels will be necessary, as outlined in Figure 1.

### Defining the Population and Intervention

The significant impact of molecular pathology on adjuvant strategies has rendered the “wait and see” approach unjustifiable (7, 20). Stereotactic biopsy is a technique familiar to all neurosurgeons and a strategy applied to DLGG management at many centers (16, 25). In some retrospective studies advocating for MSR, patients undergoing biopsy have been excluded, hindering comparisons with subtotal resections (13, 27). For supratotal resections, the data are compelling but based on small retrospective series (21, 28). Thus, there are at least two clinically important patient cohorts for which more objective evidence can be explored: (1) Individuals with tumors in regions traditionally considered eloquent with low-risk preoperative features (i.e., low Pignatti score: younger age, smaller tumor, lack of neurological deficits, etc) (29); and (2) Individuals with tumors in regions traditionally considered non-eloquent.

For the first patient cohort, individuals may be randomized to stereotactic biopsies vs. a pragmatic philosophy of debulking of any extent. Appropriate adjuvant therapy would be based on molecular and clinical features. This cohort of patients, in addition to those with tumors in eloquent areas, might also include those with diffuse tumors for which radical resection is not easily attainable. In these individuals, an RCT would be particularly informative in objectively establishing whether any surgical approach that is more aggressive than stereotactic biopsy, when radical resection cannot be attained, is superior in terms of agreed upon endpoints. This would also provide a foundation to explore emerging minimally invasive approaches of tumor sampling, such as liquid biopsies aided by blood-brain barrier disrupting technologies such as MR-guided focused ultrasound (30, 31). Conversely, technological advancements that allow access to deep-seated brain tumors with minimal disruption of white matter tracts may enable more aggressive resection (32).

In the second population cohort, supratotal resection could be compared to MSR. While the basic principle for determining whether more aggressive surgery is superior is similar to the first group, this would be a comparison of two competing philosophies of functional- vs. anatomically-guided surgery, respectively (28). This implicitly necessitates standardization of imaging modalities and algorithms along with operating procedures for intraoperative stimulation mapping (ISM).

### Standardization of Protocols

Standard MRI sequences have an important diagnostic and prognostic role in DLGG diagnosis and management, particularly with respect to identifying accurate tumor margins for resection (33). Advanced imaging sequences and modalities are increasingly incorporated into practice. Examples include perfusion MRI, diffusion tensor imaging (DTI) (34), functional MRI (resting state and task-based), MR spectroscopy (MRS) (35), and PET imaging with amino acid tracers (36). Intraoperative MRI has also been used to improve the extent of resection of LGGs when compared to traditional approaches (37). These imaging modalities have been influential in better understanding brain physiology and pathology. However, we have yet to consolidate these modalities into a standard multi-parametric imaging protocol for DLGG management. This is further compounded by the difficulty of distinguishing tumor from peritumoral edema prior to surgery, which is an ongoing difficulty with current imaging techniques (37). This issue will only become more difficult with the advent of novel molecular signatures that might reveal further heterogeneity in DLGGs.

ISM, performed systematically, is the gold-standard of comparison for functional imaging (38). Extensive analyses of brain connectivity, based on ISM data, have enabled creation of probabilistic atlases of essential cortical regions (39, 40). These are critical as seeding points in specific imaging protocols while also serving as essential guideposts for individualizing ISM. Depending on the lobe(s) affected, extent of tumor infiltration, preoperative functional deficits, and vocational needs of each particular patient, specific intraoperative cognitive and functional tasks can be used during ISM to strike the ideal oncological and functional balance of tumor resection (41). This approach is also paramount for a postoperative cognitive rehabilitation pathway (42). Data can no longer be confined and analyzed in isolation among specialties. The ISM-based functional mapping literature is robust and instructive, and correlation of emerging imaging data within this literature will generate valuable insights. Standardized algorithms can only be developed when these insights are validated for clinical relevance (Figure 1).

### Choice of Endpoint(s)

While PFS/mPFS are used in many oncology trials, ascertainment is problematic owing to the possible subjective nature of these endpoints, and in DLGGs they may not necessarily correlate with OS (4). Although more objective, the use of OS can be challenging in DLGG trials as the increasing life expectancy could affect trial feasibility. In addition, subsequent therapy following study protocol interventions may differ among patients, thereby confounding the OS in long-survival diseases such as DLGG. Given these confounding influences, quality of life, and preservation of function should be critical endpoints as well. These assessments should ideally be based on patient-reported outcomes (PROs), which should be direct and elucidate patient concerns and burden of treatment on patients (43, 44). Particular attention must be given to selecting PRO instruments validated in brain tumor patients.

Given that both OS and PROs are important to clinicians, patients, and policymakers, an acceptable approach would be to combine OS and PROs as a composite endpoint (CEP), reducing the required sample size and enabling assessment of the net benefit of an intervention (45). This, however, also presents the potential risk of one endpoint dominating this net benefit. Furthermore, the combination of fatal (OS) with non-fatal endpoints (PRO), could theoretically result in an erroneous conclusion of net benefit for a particular intervention. For example, an aggressive surgical resection may increase OS at the expense of function. Seeking input from patients and the general population regarding the value of survival and function is therefore necessary in establishing outcome thresholds (Figure 1).

### Challenges Specific to Surgical RCTs in DLGGs

Surgeon biases toward treatment allocation is a challenge in conducting surgical RCTs. In pathologies of high prevalence, this may be remedied by implementing an expertise-based trial wherein surgeons only perform the intervention proposed in one arm of the study (e.g., only MSR rather than supratotal resection for DLGG) and would therefore only manage patients randomized to that particular arm. This approach becomes challenging for conditions with low prevalence like DLGGs, as the number of centers and surgeons providing specialty care may be limited. Surgeon technical skill is another important factor to consider. Ideally, surgeons would have reached the plateau phase of the technical learning curve prior to their involvement in a trial (46). Options for evaluating technical competency may involve a minimum number of cases performed, a report of past complications, or on-site assessment of technique (47). While this approach helps increase validity, it may introduce constraints into the protocol that limit generalizability.

In contrast to drug trials where patients can only receive a novel drug in the setting of an RCT, standard surgical procedures can be performed more universally outside the confines of a clinical trial. With increasing avenues for patient data access and growing patient autonomy over treatment decisions, the chances of individuals seeking a particular intervention make recruitment into an RCT challenging (48). For surgeons, this reality is an additional disincentive for participation as they may lose their referral source. Open discussions about treatment options and the current state of the evidence will therefore be essential in setting a framework for surgical trials in DLGGs.

The extended period of enrolment and analysis required for a surgical trial for a low-frequency and long time-to-endpoint disease such as DLGG can add an extra layer of complexity. During the course of the trial, the development of better adjuvant treatments (e.g., IDH1 inhibitors) will have a differential impact on OS for patients enrolled before and after their widespread use (49). This is in addition to the high probability of drop-out over many years of follow-up, although there is precedence for extended trial durations in DLGGs (4, 5). Pragmatic RCTs which allow wide-ranging protocol flexibility after randomization may address the challenges associated with standard RCTs (50); however, consideration must be given to potential concerns regarding blinding and allocation concealment.

## Harnessing the Power of Observational Data

In light of the challenges in conducting RCTs for DLGGs and the need to develop hypotheses relevant to an “everyday” setting, international registries of prospective observational data may offer an important tool in developing evidence based management guidelines (51). Resources aimed at compiling relevant clinical information on DLGGs have already been developed (https://lggregistry.wixsite.com/study); however, these lack a systematic approach to prospective collection and annotation of data. The validity and yield of international multi-center collaborative studies in DLGGs has been demonstrated (4, 5). Furthermore, natural language processing, computational capabilities, and healthcare information technology continue to improve, making electronic health/medical records (EHR/EMR) an invaluable resource. Combining these resources may enable the compilation of prospective clinical, imaging, surgical, functional, histopathological/ molecular, and neurocognitive data into an international multi-center database for large scale analysis, allowing for more efficient and economical analysis than possible in RCTs.

Nevertheless, this approach presents its own pitfalls. Increasing legal and privacy laws may limit the inter-institutional flow of patient information (52). Moreover, a lack of data standardization between EMRs poses further challenges in efficiently combining data, and would require significant coordination and collaboration between participating centers in order to produce data that would be homogenous enough to analyze as a single data set. However, patients own the rights to their clinical information and should decide whether this information is shared (53). In conjunction, better connectivity between clinical and legal teams is necessary to devise comprehensive strategies that enable the greatest advancement of science and patient care within the confines of the law. Healthcare professionals must continue their role as patient advocates by participating in the improvement of EMRs to increase fidelity. Oversight by experts in the field would also ensure the collection of relevant data, which would help increase efficiency.

Successful establishment of international registries would enable the acquisition of large-scale multi-modal data. Using propensity score matching, known confounders could then be accounted for, enabling identification of associations and correlations that might predict success with certain treatment paradigms in a manner that most closely resembles RCTs (54). However, this would require a hypothesis-driven approach, *a priori* knowledge of potential confounders, and may result in potential exclusion of putative covariates that can be influential. Thus, alternative data-driven methods such as machine learning be required to analyse these registries.

Machine learning and artificial intelligence algorithms in general have seen significant uptake in healthcare research in recent years, and these tools show great promise for use in analyzing large datasets in LGG as well (55). The traditional method of statistical inference from data, namely regression-based prognostic modeling, loses stability when dealing with large data sets in which co-linearity between multiple variables might exist, such as in potential large-scale observational registries (56). When this occurs, some predictor variables in the data might be able to be linearly predicted by others, causing inaccurate estimates about the impact of any one variable on the outcome of interest. In addition, traditional regression models are often unreliable with datasets that contain large feature: sample size ratios, wherein large numbers of predictor variables exist for a dataset with limited sample size to draw conclusions from. Many machine-learning algorithms, on the other hand, address these issues and thereby provide greater ability to analyse the complex sets of data procured from large registries. A particularly appealing application of these algorithms to the study of DLGGs is the ability to continually “learn” and refine the model(s) created. “Supervised” algorithms, including support vector machines and artificial neuronal networks, use a “training” set of data to develop a classification algorithm, and then apply the learned algorithm to novel data sets to help identify features in new data (57). These techniques might be harnessed in the setting of DLGG by using large observational data sets to identify factors in imaging/patient characteristics that might predict response to certain modalities of treatment, and therefore inform patient care. Furthermore, “unsupervised” learning algorithms, such as hierarchical or *k*-means clustering, can be applied to identify novel patterns in imaging and molecular data through an unbiased approach, without any prior “training” data (58). This new information can then be assessed for predictive/classification potential. In the setting of DLGG, these methods might be valuable for identifying patterns in patient data and classifications of tumors that might not be otherwise immediately recognizable. It is essential to note that one algorithm or model is not suitable in all scenarios and a combination of strategies, such as “blending,” may be required (Figure 1). Table 1 provides a high-level comparison of various data analysis methods that might be of use in DLGG research, and Table 2 defines some common terms used in statistics and machine learning.

**Table 1:** Comparison of current analytical methods utilized in data-driven research with potential for application to DLGG research.

**Method**	**Advantages**	**Limitations**
**SUPERVISED LEARNING**		
Regression	• Commonly used • Easy to interpret • Low variance (high generalizability)	• Multi co-linearity • Large feature: sample size ratio • High bias (training error)
Decision trees	• Applicable to both classification and regression • Intuitive design and presentation • Applicable to various data types	• Over-fitting • Mutually exclusive classes needed • Order of decision node selection impacts results
Random forest	• Addresses issue of over-fitting in Decision Trees	• Less interpretable than Decision Trees
K-nearest neighbors	• Applicable to both classification and regression • Works well with missing data	• Predictions based on similarities rather than creating models • Assumption of equal relevance for features • Forced classification of features based on similarities
Support vector machines	• Over-fitting less likely • Robust with large features: sample size ratio • Reduced computational complexity	• More complex than Decision Trees • Hard to interpret
Naïve bayes	• Based on commonly known statistical principles • Higher classification speed • Easy to interpret	• Assumption of independence • Assumption of normal data distribution • Frequency of observations affect accuracy of model
Neural networks	• Applicable to both classification and regression • Versatility of methods (statistical and Boolean operations)	• Selection of type and combination of layers challenging • Difficult to interpret
**UNSUPERVISED LEARNING**		
Self-organizing feature maps	• Dimensionality reduction along with clustering • Easy to interpret	• Affected by missing data • High computational cost
Hierarchical clustering	• Does not require pre-specification of number of clusters • Visually easier to interpret	• Framework for selecting metrics such as linkage type and measure of dissimilarity difficult to establish
K-means clustering	• Computational efficiency	• Requires pre-specification of number of clusters • Affected by outlier data

**Table 2:** Common terms and definitions used in machine learning research.

**Term**	**Definition**
Artificial intelligence	• The development of computer algorithms and/or systems that are capable of performing tasks which traditionally require human intelligence, including visual perception, speech recognition, decision-making, and categorization
Data mining	• A multifactorial approach to identifying patterns and correlations within large datasets using statistics, machine learning, and database management software
Machine learning	• A subset of AI; computer algorithms that use a set of “training” data to identify patterns, enabling prediction of future data trends and classification of previously unseen data. ML algorithms are continually able to adjust and learn from new data to improve predictive or decision-making performance.
Natural language processing	• A subfield of AI and linguistics that uses machine learning techniques to analyse language data, including speech recognition and generation.
Deep learning	• A branch of machine learning that is based on algorithms known as artificial neural networks
Neural networks	• Computational algorithms used in machine learning that are inspired by biological neural networks; these algorithms are capable of learning to perform complex tasks by using training data to inform actions taken on new data, without specific rules needing to be hard-coded for each task
Supervised learning	• An approach to machine learning that uses labeled training data to train an algorithm to predict a desired and known output variable from new input data. The characteristics of the training data are known to the researcher.
Unsupervised learning	• An approach to machine learning that does not use any specific training data, and instead trains an algorithm on an entire set of input data. The goal is to uncover associations and structure within the data, without a known and pre-specified output variable.

Similar computational approaches have shown great promise in other areas of oncology, such as breast cancer (59). Further, international competitions to develop the most efficient computational models for predicting cancer survival have been successful in breast cancer, where the most effective crowdsourced model outperformed the previously existing best-in-class model reported in the literature when scored by blinded assessment (59). Therefore, opening the forum to competition to develop the best predictive models for DLGG by harnessing large-scale observational data sets might be a unique and useful approach.

Despite the promise of ML techniques, these algorithms are not a solution to poor data quality; poor input results in poor output. Similarly, internal and external validity must be demonstrated on an independent test dataset; this is particularly relevant to ML algorithms as small sample sizes or highly constrained models can result in over-fitting of training data (58). This issue is particularly important for LGG as they are a low-frequency set of diseases, making it difficult to procure large enough datasets to train and validate clinically relevant and robust ML models. Despite this problem, past work in developing ML models to predict glioma grading and molecular characterization based on imaging has been quite successful. For example, Akkus et al. developed an algorithm to predict deletion of chromosome arms 1p/19q from T1/T2 imaging using a cohort of 159 LGGs, with sensitivity of 93.3% and specificity of 82.2%, showing promise in using ML to inform glioma management (60, 61). These existing works are promising and showcase the immense potential for ML in future glioma research.

Ideally, any ML-based algorithms used in informing clinical decision making or evaluated in clinical trials will be declared and registered *a priori*, as is expected for clinical trials. When feasible, the data source should be made public along with the algorithms used for analysis. Consideration should be given to the development of quality checklists, such as the CONSORT statement for clinical trials, in studies reporting ML-based findings. These efforts are already underway by the CONSORT AI and SPIRIT AI steering groups, using the EQUATOR guideline development framework to develop extensions to the CONSORT and SPIRIT statements for AI-based studies (62). Once particular predictive/classification models are better established, association with outcomes and perhaps causality can be assessed through clinical trials.

## Conclusion

In this critical review, we have provided an overview of the current state of evidence pertaining to DLGG management, highlighted some of the gaps in knowledge, and outlined possible strategies for the acquisition of better evidence. As part of this endeavor, we have first discussed the nuances and challenges associated with conducting the gold standard approach, an RCT. Recognizing the difficulty in overcoming some of these challenges, we have outlined more pragmatic approaches, including more effective collection of data through large-scale registries and ML-based statistical analysis of this “real-world” observational data. As shown in Figure 1, we have outlined a strategy for utilizing modern data analysis strategies and various data sources to develop more compelling data for informing DLGG care, and have outlined a pathway for refining this strategy using feedback from the neuro-oncology community. Moving forward, it is critical for the neuro-oncology community to evaluate the necessity and specific eligibility criteria for an RCT, work to develop more effective prospective observational registries, and integrate various data sources using data-driven computational analysis, in order to develop higher quality evidence for DLGG management and ultimately improve patient care for this group of tumors.

## Data Availability Statement

The original contributions presented in the study are included in the article/supplementary material, further inquiries can be directed to the corresponding author/s.

## Author Contributions

All authors contributed to the conceptualization and editing of the article. KB and AM wrote the manuscript with significant input from all authors.

## Conflict of Interest

The authors declare that the research was conducted in the absence of any commercial or financial relationships that could be construed as a potential conflict of interest.
